# Propofol versus the world: The whole is not always greater than the sum of its parts

**DOI:** 10.1186/s13054-023-04450-5

**Published:** 2023-05-30

**Authors:** Jan Hansel

**Affiliations:** University of Manchester, Manchester, UK

Dear Editor,

I read with interest the recently published systematic review and meta-analysis update by Kotani et al. [1]. The findings of their work, suggesting an increased mortality associated with propofol (RR 1.10, 95% CI 1.01–1.20, *p* = 0.03) appear to reverse those of the original review from 2015 [2], where no difference in mortality was demonstrated between patients receiving propofol or any other comparator (RR 1.05, 95% CI 0.93–1.18, *p* = 0.5).

Firstly, the authors should be congratulated on the considerable methodological effort that will have gone into the conduct of such a large systematic review, including a welcome Bayesian meta-analysis and embedded trial sequential analysis. I will forego belaboring the usual criticisms aimed at evidence synthesis research, such as the considerable clinical heterogeneity of combining studies from various environments and different exposure periods to the agents in question, and would instead rather focus on what may be driving this difference in estimates.

The authors correctly identified several subgroups where effects were more pronounced (studies looking at cardiac surgery, adults, comparing propofol to volatiles, among others), which they further elaborate on in the discussion section. However, they stop short of addressing the elephant in the room. Looking at Figure S4 (forest plot for cardiac surgery as the setting), only one large study does not cross the line of no effect [3]. It appears that Kotani et al. extracted data for 1 year mortality, even though the study by Likhvantsev et al. also reported a much less sensational effect estimate for 30-day mortality. This is at odds with Kotani’s PROSPERO registration (CRD42022323143), although the authors reference a change in protocol following data extraction. It would have been more appropriate to extract mortality closest to the initially planned 30-day mortality, if reported. Furthermore, it appears that they extracted as intention-to-treat (*n* = 450 per arm as the denominator), where it would have been more appropriate to extract the number of patients for which follow-up data were available. This will have further artificially inflated the estimates (see Fig. 1). Finally, the choice of a fixed-effect model is questionable given the considerable underlying clinical heterogeneity; a random-effects model would have been more appropriate [4].

**Fig. 1:**
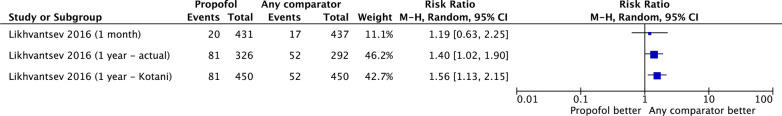
Comparison of effect estimates depending on data extraction decisions. The top extraction would be consistent with the protocol specifications. The bottom extraction of one year mortality estimates uses incorrect denominator data. Risk ratios are presented using a Mantel–Haenszel random-effects model, as opposed to a fixed-effect model. Note that this plot was not created with the intention of pooling, but rather to compare: therefore, the summary estimates have been removed

I believe readers of *Critical Care* would appreciate a re-analysis of the data, especially as the estimates reported in the abstract summary may be the consequence of erroneous data extraction. I suspect such a re-analysis will significantly impact the overall pooled findings of the meta-analysis presented by Kotani et al.

## Data Availability

No datasets were created during the production of this work.
